# Probiotic Bacterium-Derived p40, p75, and HM0539 Proteins as Novel Postbiotics and Gut-Associated Immune System (GAIS) Modulation: Postbiotic-Gut-Health Axis

**DOI:** 10.3390/microorganisms13010023

**Published:** 2024-12-26

**Authors:** Feray Gençer Bingöl, Duygu Ağagündüz, Ferenc Budán

**Affiliations:** 1 Department of Nutrition and Dietetics, Faculty of Health Science, Burdur Mehmet Akif Ersoy University, 15200 Burdur, Türkiye; fgencer@mehmetakif.edu.tr; 2 Department of Nutrition and Dietetics, Faculty of Health Science, Gazi University, 06490 Ankara, Türkiye; duyguturkozu@gazi.edu.tr; 3 Institute of Physiology, Medical School, University of Pécs, H-7624 Pécs, Hungary

**Keywords:** p40, p75, HM0539, postbiotic protein, gut-associated immune system

## Abstract

It is known that probiotics have direct and indirect effects on many systems in the body, especially the gastrointestinal system. Interest in using probiotic strain-derived cell components and metabolites has also increased as a result of the significant benefits of probiotics. Although many terminologies and definitions are used for these components and metabolites, the International Scientific Association of Probiotics and Prebiotics (ISAPP) recommended the use of the term postbiotic in 2021, which is defined as “a preparation of inanimate microorganisms and/or their components that confers a health benefit on the host”. Postbiotics are bioactive metabolites such as organic acids, peptides/proteins, cell wall components, functional enzymes, short-chain fatty acids, vitamins, and phenols. These molecules mediate many positive effects such as immunomodulatory, antimicrobial, and antioxidant effects. These positive effects on maintaining health have enabled the identification of many new postbiotic proteins such as p40, p75, and HM0539. In this review, the postbiotic proteins p40, p75, and HM0539 derived from lactobacilli and their functional effects are systematically summarized. The p40 protein, in particular, has been shown to support gut barrier activity and reduce inflammation, potentially through mechanisms involving epidermal growth factor receptor-dependent signaling. Additionally, p40 and p75 proteins exhibit protective effects on intestinal epithelial tight junctions, suggesting their therapeutic potential in preventing intestinal damage and diseases such as colitis. HM0539 enhances intestinal barrier integrity, exhibits antiinflammatory properties, and protects against bacterial infection, suggesting its possible as a therapeutic for inflammatory bowel disease. This review may contribute to future studies on the therapeutic use of p40, p75, and HM0539 postbiotic proteins in inflammatory gastrointestinal system diseases.

## 1. Introduction

The intestine is an organ colonized by a large number of microorganisms called microbiota. The mutualistic relationship between humans and microbiota has given humans functions that have a significant impact on biological processes like nutrient utilization, detoxification, metabolic regulation, and immune system control [1,2,3]. Due to these critical roles of the intestinal microbiota in living organisms, it is important to maintain healthy microflora. This situation has increased the visibility of probiotics, defined as “live microorganisms that, when administered in adequate amounts, confer a health benefit on the host”, in microbiota regulation [4]. In addition to probiotics, the effects of postbiotics, defined as “preparations of inanimate microorganisms and/or their components that confer a health benefit on the host” on the microbiota are also being evaluated [5]. Postbiotics are thought to be effective for both intestinal health and the gut-associated immune system (GAIS) by interacting with the immune and neurological systems, strengthening the innate immune system function, reducing pathogen-induced inflammatory responses, and improving intestinal epithelial barrier function [6,7]. Organic acids, peptides/proteins, enzymes, bacterial lysates, short-chain fatty acids, vitamins, phenols, exopolysaccharides, and cell wall components are known examples of postbiotic molecules [7,8,9].

Studies confirming the specific effects of postbiotics on maintaining health have also shed light on the identification of new postbiotics [10,11,12]. Proteins p40 and p75 are cell wall proteins that have been reported in several lactobacilli species, including *Lacticaseibacillus casei, Lbs. paracasei*, and *Lbs. rhamnosus*. p40 and p75 proteins have shown a crucial effect in modulating intestinal epithelial cell survival and function [13,14]. p40 has been extensively studied and demonstrated to possess several beneficial properties such as the inhibition of apoptosis, the enhancement of mucin production, and the promotion of immunoglobulin A (IgA) secretion. Although p75 has been studied less than p40, it has often been studied in conjunction with p40 to see its effects on gut health. Collectively, p40 and p75 represent a subset of postbiotic proteins with promising potential for GAIS [15,16,17].

Another new postbiotic, HM0539, was identified as a soluble protein secreted by *Lbs. rhamnosus* GG [18]. While the precise mechanisms of action are still under investigation, accumulating evidence suggests its involvement in the immunomodulation and modulation of inflammatory responses. Studies indicate that HM0539 may interact with host immune cells and signaling pathways, potentially influencing the cytokine production of and other inflammatory mediators [18,19].

The capabilities of p40, p75, and HM0539 to modulate intestinal epithelial cell survival and function, inhibit inflammation, and enhance mucosal immunity make them attractive candidates for further research and potential therapeutic applications in various gastrointestinal and immune-related disorders [20]. This review aims to present current evidence on the physiological effects of p40, p75, and HM0539 proteins, novel postbiotic proteins derived from probiotic bacteria, on GAIS and the system of their interaction with host cells.

## 2. Gut-Associated Immune System (GAIS)

The gut-associated immune system is a highly specialized component of the immune system that plays an essential role in controlling gut homeostasis and immune function. This system mediates a delicate balance between tolerance to dietary antigens and commensal microbiota and defense against pathogenic organisms. The GAIS is primarily represented by the gut-associated lymphoid tissue (GALT), epithelial barrier, innate and adaptive immune cells, and microbial interactions [21,22,23].

The GALT includes Peyer’s patches, isolated lymphoid follicles, mesenteric lymph nodes, intraepithelial lymphocytes, and lamina propria. Peyer’s patches are organized lymphoid follicles located in the ileum that contain a high density of B cells, T cells, macrophages, and dendritic cells. Peyer’s patches host functional epithelial cells called microfold cells that facilitate the transition of antigens from the intestinal line to the underlying lymphoid tissue [24]. Isolated lymphoid follicles, like Peyer’s patches, are distributed throughout the small and large intestines and are involved in B cell responses, particularly IgA production [25]. Intraepithelial lymphocytes contain subsets of T cells that stimulate innate and adaptive immune responses to provide protection at epithelial barrier sites [26]. Lamina propria is a connective tissue underlying the intestinal epithelium, populated with immune cells. The immune cells it contains are crucial for regulating local immunity [27].

The intestinal epithelial barrier presents as the primary line of defense against gastrointestinal pathogens, toxins, and other adverse substances. The intestinal epithelial barrier is a complex structure composed of functional epithelial cells, tight junctions, a mucus layer, immune cells, and an extracellular matrix. Intestinal epithelial cells include enterocytes for nutrient absorption and barrier function, goblet cells for mucus secretion, Paneth cells for antimicrobial peptide production, and enteroendocrine cells for hormone secretion. The tight junctions are composed of several transmembrane proteins, including claudins, occludin, and zonula occludens proteins. These proteins regulate paracellular permeability, ensuring that only selective substances can pass between cells while preventing the entry of toxins and infections. The mucus layer, produced primarily by goblet cells, serves as a physical barrier covering the epithelial surface. This layer also contains secretory IgA and antimicrobial peptides that contribute to immune defense. Together, these components work synergistically to protect against pathogens. This barrier integrity is critical for overall gut health and preventing inflammatory diseases [28,29,30,31].

Macrophages, dendritic cells, and innate lymphoid cells, which are part of the GAIS infrastructure, contribute to the innate immune reaction. These cells respond to pathogens while maintaining tolerance to commensal bacteria [32]. The adaptive immune system acts as a secondary defense mechanism against pathogens. B cell-mediated humoral immunity and T cell-mediated cellular immunity are components of the adaptive immune system. By secreting IgA antibodies, adaptive humoral immune mediators such B cells preserve intestinal homeostasis. T cells are differentiated to create an immune response to changes in the intestinal environment in cellular immunity [33].

It is known that intestinal microbiota interacts with the GAIS and affect systemic immune activity and the functional integrity of the intestinal barrier [34]. The effect of intestinal microbiota appears with different mechanisms in the development of both innate immunity and adaptive immunity. For instance, the production of mucin, the barrier component, can be impaired in conditions such as the presence of pathogens and malnutrition. This condition is associated with the onset of inflammatory bowel diseases [35,36]. Numerous biologically active metabolites secreted by the intestinal microbiota are also known to be associated with the immune system. For instance, short-chain fatty acids, which are postbiotics, have a regulating effect on the adaptive immune system [37]. GAIS is a complex and dynamic system for preserving gut balance and protecting the host from pathogens. The complicated network of lymphoid structures and immune cells enables effective immune control, appropriate immune responses, and the maintenance of resistance to harmless antigens [38].

## 3. Postbiotic Proteins: Multifunctional Mediators of GAIS Modulation

Postbiotic proteins are thought to be effective for GAIS due to typical structures, such as reducing inflammatory responses and strengthening the intestinal epithelial barrier [39]. The main postbiotic proteins are cell surface proteins. Cell surface proteins are crucial constituents of the outmost cell envelope structures seen on the surface of lactobacilli and other probiotic bacteria species. The LPxTG protein is a significant transmembrane protein that exhibits high hydrophilicity, stability, and adhesion capacity. It was observed that the LPxTG protein with esterase activity supports the adhesion of lactic acid bacteria to HT-29 cells by binding to the annexin A2 protein on the cell surface of the target cells in addition to esterase activity. In addition, it enabled the hydrolysis of lipids and the release of polyunsaturated fatty acids in the intestine through esterase activity. These multifunctional properties of LPxTG proteins have been shown to play critical roles in bacteria–host interactions [40].

The surface layer (S-layer) is a glycoprotein-based cell surface layer found in several bacterial species, serving as a two-dimensional macromolecular component that covers the cell surface. Both pathogenic and non-pathogenic types of bacteria have been shown to contain the S-layer [41]. S-layer proteins identified in probiotic bacteria have also been shown to prevent pathogen infection by competitively binding to the extracellular matrix and host cell proteins. These proteins act on the immune system by interacting with specific receptors on the surface of different cells that trigger proinflammatory or antiinflammatory responses. The S-layer proteins have been shown to exhibit a range of immunomodulatory properties, influencing both innate and adaptive immune responses. These proteins influence innate immunity through pattern recognition receptor activation, mucosal barrier function, and antimicrobial activity. The S-layer proteins contribute to the production of cytokines by interacting with pattern recognition receptors such as toll-like receptors produced by cells of the host immune system. Moreover, cell integrity and permeability were also improved after S-layer proteins stimulation by regulating the expression of tight junction proteins such as zonula occludens-1 and occludin [42]. Also, some S-layer proteins can prevent dysbiosis by inhibiting the growth of pathogenic bacteria [43]. The S-layer proteins act on adaptive immunity by promoting the differentiation of T regulatory cells (Tregs) and by promoting IgA production via the B cell response. In addition, these proteins support the antiinflammatory response by regulating the production of cytokines such as interleukin-10 and interleukin-12, which play an important role in the regulation of immune responses [44,45,46].

Pili are elongated protein structures that were initially discovered through the use of electron microscopy in Gram-negative bacteria during the early 1950s. In recent years, pili proteins have also been found in many Gram-positive bacteria, including Streptococcus and Enterococcus pathogens [47]. The existence of mucus-binding pili on the surface of a non-pathogenic Gram-positive bacterial strain, *Lbs. rhamnosus*, has shown that these proteins are not unique to pathogens [48]. These protein structures extending from the bacterial surface consist of pilin subunits that play an essential role in adhesion and host colonization [47]. It has been reported that pili structures increase the adherence time in the gastrointestinal tract compared to lactobacilli without pili, with both long-range and close-range contact and mucus binding power [49]. It has also been found that pili structures play a critical role in the adhesion capacity of macrophages, support bacterial uptake by phagocytic cells, and mediate antiinflammatory effects through the induction of interleukin-10 mRNA and the reduction in interleukin-6 mRNA in the macrophage cell line [50].

Moonlighting proteins perform multiple physiologically relevant biochemical or biophysical functions. Moonlight proteins found in bacteria include several classes of proteins, including metabolic enzymes and molecular chaperones. They differ from other postbiotic proteins in that they do not contain a surface attachment or a secretion signal. Moonlight proteins are thought to enhance adhesion and colonization to the host epithelium, as well as regulate host components such as extracellular matrices and plasminogen and the host immune response [51,52].

Some protein structures secreted by probiotic bacteria have also been observed to have postbiotic effects. The aggregation-promoting factor has been identified as an extracellular secretory protein involved in the conjugation and autoaggregation of lactobacilli. The aggregation-promoting factor from lactobacilli bacteria appears to be associated with the immune system by promoting host colonization and the exclusion of pathogens [39].

Bacteriocins are ribosomally synthesized antimicrobial peptides produced by both Gram-positive and Gram-negative bacteria. Bacteriocins from Gram-positive bacteria are small peptides, usually 30–60 amino acids long. These peptides are generally heat-stable and exhibit self-protective mechanisms against their antibacterial effects [53]. Lantibiotics are the most extensively modified group of bacteriocins containing unique amino acids [54]. Bacteriocins from Gram-positive bacteria broadly show this function by forming pores or disrupting the cytoplasmic membrane [55]. These peptides show antimicrobial activity against related species and pathogens like Salmonella, Staphylococcus, Listeria, Clostridium, and Enterococcus. They are also known to be effective against viral infections such as rotavirus, norovirus, and adenovirus. Probiotics and their bacteriocins regulate the intestinal microbiota to keep the host immune system in balance through antimicrobial effects and immune modulation [56]. Bacteriocins from Gram-negative bacteria produce a different set of bacteriocins, including larger proteins known as colicins (>10 kDa) and smaller peptides called microcins (<10 kDa) [54]. Colicins exhibit a narrow spectrum of activity and primarily target *E. coli* strains and closely related Gram-negative bacteria. The action mechanism of colicins typically involves the disruption of the membrane integrity of the target cell or interference with basic cellular processes such as DNA replication or protein synthesis through specific receptor-mediated uptake. Microcins, on the other hand, exhibit a broader range of activity against a variety of Gram-negative bacteria. This bacteriocin is generally more resistant to proteolytic degradation, changing pH levels, and extreme environmental conditions. The action mechanism of microcins involves pore formation in the target membrane or the inhibition of specific metabolic pathways. The synergistic approach of combining these bacteriocins (colicins and microcins) with antimicrobial agents is considered promising for increasing their efficacy against resistant Gram-negative pathogens and reducing the impact of antibiotic resistance in clinical settings [57,58].

### 3.1. Protein p40 and p75

Although the positive effects of probiotics on the intestinal microbiota are known, the underlying mechanisms are still being elucidated. Recently, it has been observed that some probiotic-derived protein-structured postbiotic components such as bacteriocins, cell surface proteins, and secreted proteins have similar positive effects to live probiotics. New ones are added to these postbiotic proteins every day. Yan et al. reported in 2007 that two proteins (p40 and p75) purified from *Lbs. rhamnosus* GG stimulated the activation of Akt, a signal transduction pathway that promotes survival and growth in cultured cells and exvivo colon organ culture models, promoted cell growth, and inhibited tumor necrosis factor-induced epithelial cell apoptosis. These two proteins are named p40 and p75 because their molecular masses are approximately 40 kilodaltons and 75 kilodaltons [13]. Bäuerl et al. showed in 2010 that genes encoding homologues of p40 and p75 proteins are also present in other closely related strains of the *Lbs. casei/paracasei/rhamnosus* group. The researchers reported that p40 and p75 proteins were secreted by *Lbs. casei* BL23 and located on the bacterial cell surface [59]. Bäuerl et al. showed in 2019 that both p40 and p75 proteins are also present in *Latilactobacillus sakei* phylogenetic groups as a result of phylogenetic analyses [60].

When the functions of these proteins were examined, it was reported that the p40 protein decreased intestinal epithelial apoptosis and barrier activity impairment in the colonic epithelium in dextran sulphate sodium-induced experimental colitis in an epidermal growth factor receptor-dependent manner [16]. It has been stated that p40 contributes to the support of IgA production by increasing the production of epidermal growth factor receptor-dependent proliferation-inducing ligand (APRIL) to prevent intestinal inflammation in intestinal epithelial cells [15,61]. In addition to reducing intestinal inflammation, p40 production increased under conditions with healthy colonic lumen content. This has been attributed to the promotion of postbiotic p40 production by heat shock protein 90 (HSP90) [62]. On the other hand, p40 has been shown to increase epithelial barrier function through different signaling pathways, such as increasing transepithelial electrical resistance and the expression of the tight binding protein occluding [14]. It has been shown that p40 supplementation in mice during the neonatal period induces the long-term epigenetic repression of transforming growth factor-β (TGF-β) by regulating methyltransferase activity in intestinal epithelial cells. This mechanism suggested that p40 activity in TGF-β production in early life may be a preventive factor for adult colitis [63]. Similarly, neonatal p40 supplementation has been reported to protect against intestinal injury and colitis by promoting protective immune responses such as regulatory T cell differentiation and IgA production in adult mice [64]. Regulski et al. showed that p75 is an endopeptidase that plays a key role in daughter cell separation and is the major peptidoglycan hydroxylase of *Lbs. casei* BL23 [65]. In addition, p40 and p75 proteins have been shown to protect intestinal epithelial tight junctions and barrier activity from hydrogen peroxide-induced intestinal damage via a protein kinase C and mitogen-activated protein kinase-dependent mechanism [66]. The source and functional aspects of postbiotic proteins p40 and p75 are shown in Table 1.

### 3.2. Protein HM0539

He et al. reported in 2017 that the culture supernatant of *Lbs. rhamnosus* GG promoted the development of neonatal intestinal defenses and offered protection to neonatal rats against oral *Escherichia coli* K1 infection. It has been reported that the adhesion, invasion, and translocation of *Escherichia coli* K1 to the intestinal epithelial barrier model are prevented through the regulation of mucin production and the preservation of intestinal integrity. As a result, it was stated that the *Lbs. rhamnosus* GG culture supernatant has a prophylactic effect against systemic *Escherichia coli* K1 infection in newborns, but studies determining the specific active substances that play a role in the observed positive effect are ongoing [67]. In 2019, these researchers identified HM0539 as a secreted protein included in the positive effect of *Lbs. rhamnosus* GG culture supernatant using liquid chromatography–tandem mass spectrometry analysis. According to their findings, recombined and purified HM0539 protects the intestinal barrier by boosting the expression of intestinal mucin and avoiding damage to the intestinal barrier caused by lipopolysaccharide or tumor necrosis factor-α (TNF-α). In addition, HM0539 has been shown to have the potential to prevent colitis, lipopolysaccharide/D-galactosamine-induced bacterial translocation, and liver damage [18]. Another study focused on the antiinflammatory effect of HM0539 on colitis and its potential molecular mechanisms. The results of this study showed that HM0539 suppressed the expression of cyclooxygenase-2 and inducible nitric oxide synthase by reducing the activation of the compatible promoters, which in turn suppressed the generation of prostaglandin E2 and nitric oxide. It has also been reported that HM0539 can modulate distal NF-kB activation by reducing toll-like receptor-4 (TLR4) activation and suppressing the transduction of myeloid differentiation primary response 88 (Myd88). With these effects, it has been stated that HM0539 has promising potential for use as an antiinflammatory agent for inflammatory bowel diseases [19].

**Figure 1:**
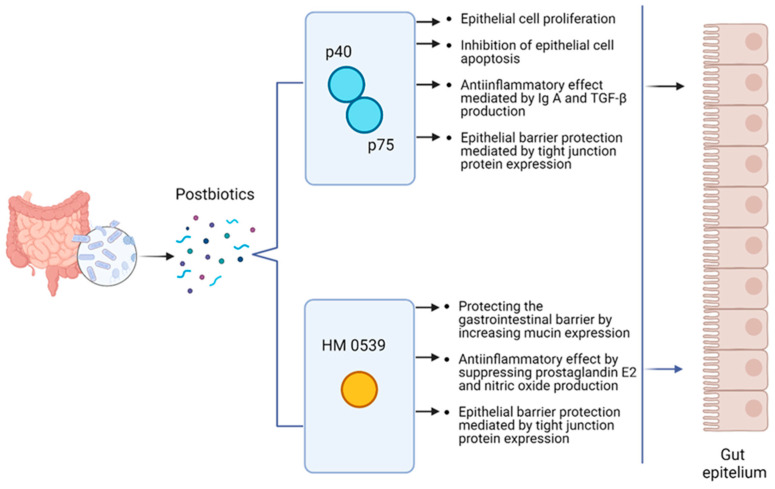
Schematic representation of various health effects of p40, p75, and HM0539 in the host. Created with BioRender.com.

In order to evaluate the inhibitory effect of HM0539 on *Escherichia coli* O157: H7 in intestinal infection, evaluations were made regarding the adhesion rate of this pathogenic bacterium to HT-29 cells. The results showed that HM0539 essentially inhibited the adhesion and invasion of *Escherichia coli* O157: H7 onto HT-29 cells. Furthermore, it was noted that the *Escherichia coli* O157: H7-induced downregulation of tight junction proteins (zonulin-1 and occludin) in HT-29 cells was alleviated by HM0539. In addition, while intestinal tissue deterioration and the infiltration of inflammatory cells in the mucosal layers were observed in mice infected with *Escherichia coli* O157: H7, mice exposed to HM0539 were observed to have relatively more normal intestinal tissues compared to the other group [68,69]. The source and functional aspects of postbiotic protein HM0539 are shown in Table 2. The immunomodulatory effect mechanisms of the current postbiotic proteins p40, p75, and HM0539 are given in Figure 1.

## 4. Conclusions

Postbiotics obtained from lactobacilli consist of a wide variety of molecular structures. It has been observed that p40 and p75 proteins stimulate Akt activation, a signaling pathway that supports survival and growth in the intestine and thus supports epithelial cell growth and inhibits epithelial cell apoptosis. It has been found to have important functions including providing antiinflammatory effects by supporting IgA and TGF-β production, as well as protecting the epithelial barrier function by increasing tight binding protein expression. Another postbiotic protein, HM0539, appears to protect the gastrointestinal barrier from pathogens and toxins by increasing intestinal mucin expression, improve gut barrier function by increasing tight binding protein expression, and exhibit antiinflammatory properties by suppressing prostaglandin E2 and nitric oxide production. All these results support the potential positive effects of natural bacterial components of novel postbiotics p40, p75, and HM0539 proteins in preventing inflammatory gastrointestinal damage and disease. Future studies on intestinal diseases, especially ulcerative colitis, Crohn’s disease, colon cancer, chronic diarrhea, and necrotizing enterocolitis, will shed light on the therapeutic applications of these postbiotic proteins.

## Figures and Tables

**Table 1:** Source and functional aspects of p40 and p75.

Bacteria	Derived Postbiotics	Functional Effects	Disease/Indication	References
*Lbs. rhamnosus* GG	p40, p75	Inhibition of cytokine-induced epithelial cell apoptosis and promotion of cell growth in colonic epithelial cells	Mouse colon epithelial cells and cultured colon explants	[13]
*Lbs. rhamnosus* GG	p40, p75	Protect the intestinal epithelial tight junctions and the barrier activity	Caco-2 cell monolayers hydrogen peroxide-induced in disruption	[66]
*Lbs. casei* BL23	p40, p75	Stimulation of epidermal growth factor receptor phosphorylation	Ex vivo mouse colon organ culture	[59]
*Lbs. rhamnosus* GG	p40	Reduction in intestinal epithelial apoptosis and disruption of barrier activity in the colon epithelium	Dextran sodium sulfate-induced experimental colitis	[16]
*Lbs. casei* BL23	p75	p75 was identified as one of the major peptidoglycan hydrolases in *Lbs. casei* BL23		[65]
*Lbs. rhamnosus* GG	p40	Contributing to the promotion of IgA production through the upregulation of APRIL expression	Mouse small intestine epithelial cells	[15]
*Lbs. rhamnosus* GG	p40	Decreased susceptibility to intestinal injury and inflammation/enhanced intestinal functional maturation and IgA production	Adult mice colonized with *Lbs. rhamnosus* GG-dextran sodium sulfate-induced experimental colitis	[61]
*Lbs. rhamnosus* GG	p40	Supporting the development of gut functions and innate immunity through EGFR	Neonatal supplementation with p40-small intestine and colonic epithelial cells	[64]
*Lbs. rhamnosus* GG	p40, p75	p40—Amelioration of intestinal inflammation p75—Less of an effect on controlling intestinal epithelial cellular response than p40	Young adult mouse colonic/small sintestinal/immortalized stomach epithelial cells	[62]
*Lbs. casei/paracasei/rhamnosus*	p40, p75	The structure, physiological function, and phylogenetic and genetic features of these postbiotic proteins have been studied.		[60]
*Lbs. rhamnosus* GG	p40	Antiinflammatory effect by providing production of TGFβ by intestinal epithelial cells and expanding Tregs	Neonatal supplementation with p40-young adult mouse colonic epithelial cells	[63]
*Lbs. rhamnosus* GR-1	p40	Increased epithelial barrier function	Cultures of the HaCaT epithelial cell line subjected to *E. coli* lipopolysaccharide	[14]

**Table 2:** Source and functional aspects of HM0539.

Bacteria	Derived Postbiotics	Functional Effects	Disease/Indication	References
*Lbs. rhamnosus* GG	HM0539	Protecting against TNF-α or lipopolysaccharide-induced intestinal barrier injury by increasing intestinal mucus expression	Neonatal rat model with *E. coli* K1 infection	[18]
*Lbs. rhamnosus* GG	HM0539	Antiinflammatory effect Inhibition of prostaglandin E2 and nitric oxide production Modulation of the TLR4/Myd88/NF-kB axis signaling pathway	Lipopolysaccharide-stimulated RAW 264.7 macrophages and, in dextran sulfate sodium-induced murine colitis	[19]
*Lbs. rhamnosus* GG	HM0539	Inhibition of *E. coli* O157: H7 adhesion and invasion to HT-29 cells Alleviation of the down-regulation of tight junction proteins Reduced *E. coli*-induced mortality and weight loss	HT-29 cells	[68,69]

## Data Availability

No new data were created or analyzed in this study.
